# Gemigliptin, a DPP4 inhibitor, ameliorates nonalcoholic steatohepatitis through AMP-activated protein kinase-independent and ULK1-mediated autophagy

**DOI:** 10.1016/j.molmet.2023.101806

**Published:** 2023-09-20

**Authors:** Youngmi Song, Hyekyung Yang, Juhee Kim, Yoonjin Lee, Sung-Ho Kim, In-Gu Do, Cheol-Young Park

**Affiliations:** 1 Medical Research Institute, Kangbuk Samsung Hospital, Sungkyunkwan University School of Medicine, Seoul, South Korea; 2 Division of Endocrinology and Metabolism, Department of Internal Medicine, Kangbuk Samsung Hospital, Sungkyunkwan University School of Medicine, Seoul, South Korea; 3 LG Chem Life Sciences, Gangseo-gu, Seoul, South Korea; 4 Department of Pathology, Kangbuk Samsung Hospital, Sungkyunkwan University School of Medicine, Seoul, South Korea

**Keywords:** Gemigliptin, DPP-4 inhibitor, ULK-1, Autophagy, NASH, Inflammasome

## Abstract

**Objective:**

Abnormal autophagic function and activated inflammasomes are typical features in the liver of patients with non-alcoholic steatohepatitis (NASH). Here, we explored whether gemigliptin, a dipeptidyl peptidase 4 (DPP4) inhibitor for treatment of type 2 diabetes, can induce autophagy and regulate inflammasome activation as a potential NASH treatment independent of its anti-diabetic effect.

**Methods:**

Expression analysis was performed using human liver samples obtained from 18 subjects who underwent hepatectomy. We explored the function and mechanism of gemigliptin using a methionine- and choline-deficient diet (MCD)-induced NASH mouse model and HepG2 cells cultured in MCD-mimicking medium.

**Results:**

Autophagy was suppressed by marked decreases in the expression of ULK1 and LC3II/LC3I ratio in human NAFLD/NASH patients, a NASH mouse model, and HepG2 cells cultured with MCD-mimicking media. Surprisingly, we found that the expression of p-AMPK decreased in liver tissues from patients with steatosis but was restored in NASH patients. The expression of p-AMPK in the NASH mouse model was similar to that of the control group. Hence, these results indicate that autophagy was reduced in NASH via an AMPK-independent pathway. However, gemigliptin treatment attenuated lipid accumulation, inflammation, and fibrosis in the liver of MCD diet–fed mice with restoration of ULK1 expression and autophagy induction. In vitro, gemigliptin alleviated inflammasome activation through induction of ULK1-dependent autophagy. Furthermore, gemigliptin treatment upregulated ULK1 expression and activated AMPK even after siRNA-mediated knockdown of AMPKα1/2 and ULK1, respectively.

**Conclusions:**

Collectively, these results suggest that gemigliptin ameliorated NASH via AMPK-independent, ULK1-mediated effects on autophagy.

## Highlights

• The expression of p-AMPK was restored in NASH patients and NASH mouse models.
• The expression level of ULK1 was reduced in liver tissues from NASH patients, NASH mouse models and in vitro model of NASH.
• Gemigliptin ameliorated NASH through reduction of inflammsome activation by ULK1-mediated autophagy induction.
• Our findings provide promising therapeutic strategies to ameliorate NASH caused by impairment of ULK1-mediated autophagy.

## 1 Introduction

The spectrum of nonalcoholic fatty liver disease (NAFLD) ranges from simple hepatic steatosis to nonalcoholic steatohepatitis (NASH), liver fibrosis, cirrhosis, and hepatocellular carcinoma [1]. NAFLD is the most common chronic liver disease worldwide and is receiving increased attention due to dramatic increases in obesity, diabetes, and metabolic syndrome [2]. The accumulation of triglycerides in hepatocytes results in simple hepatic steatosis, and approximately 10–30% of patients with hepatic steatosis will eventually develop NASH, which is characterized by steatosis with inflammation, ballooning of hepatocytes, and fibrosis [3,4]. NASH often leads to fibrosis, which can progress to more advanced and irreversible forms of liver disease, such as cirrhosis and hepatocellular carcinoma. Patients with type 2 diabetes not only have a higher prevalence of NAFLD, but also have more severe forms of the disease, including NASH and fibrosis. Thus, obesity and insulin resistance represent the main risk factors for NAFLD [[5], [6], [7]]. Despite advances in the understanding of NAFLD pathogenesis, the mechanisms responsible for the development and progression of NASH are unclear, and treatment options are very limited [8].

Autophagy is a process of degrading excess or damaged organelles (e.g., mitochondria, endoplasmic reticulum, and peroxisomes) or other cellular components such as lipid droplets via lysosomes; this process leads to removal of damaged organelles and protection and repair of cells from injury [9,10]. Recent studies have indicated that autophagy is a key regulator of insulin sensitivity, inflammation, and fibrosis as well as lipid turnover in the liver, and growing evidence has shown that impaired autophagy activity contributes to NAFLD progression to NASH [[11], [12], [13], [14], [15]]. Additionally, several interventions or clinical drugs used to alleviate NAFLD or known to affect the incidence of NASH have been demonstrated to alter autophagy level [[16], [17], [18], [19], [20]]. Thus, the potential benefits of autophagy in preventing steatosis, hepatocellular injury, and inflammation suggest that increasing autophagy may be an effective therapeutic strategy for NAFLD/NASH.

Dipeptidyl peptidase 4 (DPP4, also known as CD26) is a ubiquitously expressed enzyme in many organs including kidney, liver, pancreas, and placenta as well as in the circulation system as a soluble form [21]. Previous studies have reported that hepatic DPP4 expression is significantly increased in patients with NAFLD and NASH compared with healthy subjects [22,23]. In addition, DPP4-deficient rats have markedly decreased hepatic fat with lower levels of hepatic pro-inflammatory and pro-fibrotic cytokines compared with levels in wild-type animals [24]. These results suggest that DPP4 is highly related to the development of NAFLD and may be a novel biomarker for liver disease. Several recent animal studies have shown that DPP-4 inhibitors ameliorate hepatic steatosis in an animal model of type 2 diabetes and obesity [[25], [26], [27]]. These effects involve improvement of metabolic parameters that contribute to disease pathogenesis, such as improving insulin sensitivity and glycemic control. However, the potential benefits of DPP4 inhibitors on NASH, particularly on hepatic inflammation and fibrosis other than hepatic steatosis, are poorly understood. Therefore, in the present study, we investigated the potential protective effects of gemigliptin, a potent and selective DPP4 inhibitor, on NASH.

## 2 Materials and methods

### 2.1 Patient samples

Human liver tissue samples were obtained from 18 subjects (normal subjects: n = 6, steatosis subjects: n = 6, NASH subjects: n = 6) who underwent hepatectomy. Background liver slides were reviewed and scored using the NAFLD activity score (NAS) developed by the NASH Clinical Research Network. This score is defined as the unweighted sum of the scores for steatosis (0–3), hepatocyte ballooning (1–2), and lobular inflammation (0–3), ranging from 0 to 8. The score thresholds of <3 and ≥ 5 correlate with the diagnosis of not-NASH and NASH, respectively [28,29]. Staging based on the grade of fibrosis was evaluated as previously described [28]. Table S1 showed the information of all the human samples. All the human samples were histologically reviewed by an expert liver pathologist. This human study was approved by the Institutional Review Board of Kangbuk Samsung Hospital (IRB No. 2022-11-047), which exempted the need for informed consent.

### 2.2 Animal procedures

Eight-week-old male C57BL/6NCrj wild-type (WT) mice were obtained from Orient Bio Inc. and acclimated for at least one week (22 ± 3 °C, 12-h light/12-h dark cycle) before analysis. Nine-week-old male mice were separated into three groups; 1) control diet–fed group (Dyets, PA, USA); 2) methionine/choline-deficient (MCD) diet–fed group (Dyets, PA, USA); and 3) MCD diet supplemented with gemigliptin (LC15-0444),3S)-3-amino-4-(5,5-difluoro-2-oxopiperidino)-1-[2,4-di(trifluoromethyl)-5,6,7,8 tetrahydropyrido [3,4-d]pyrimidin-7-yl]butan-1-one group (LG Life Sciences Co., Ltd., Korea). In some experiments, the food was supplemented with 0.13% (w/w) gemigliptin. Food intake and non-fasting blood glucose level were measured weekly. Body weight was evaluated twice a week. After three weeks, animals were sacrificed following an overnight fast; blood and tissue samples were collected, and liver tissues were weighed. All experimental procedures were carried out in accordance with institutional protocols and were approved by the LG Life Sciences Institutional Animal Care and Use Committee.

### 2.3 Cell culture and drug treatments

Hepatocellular carcinoma HepG2 cells were grown in Dulbecco's modified Eagle's medium (Thermo FisherScientific, SH30243.01) containing 10% fetal bovine serum (Thermo Fisher Scientific, SH30071.03) and antibiotics (100 U/ml penicillin and 100 μg/ml streptomycin, Thermo Fisher Scientific, SV30010) in a 5% CO_2_ incubator at 37 °C. Gemigliptin was dissolved in dimethyl sulfoxide before dilution in culture medium. Cells were incubated with either DMEM or MCD-DMEM without FBS after pretreatment with 2 μM gemigliptin for 6 h.

### 2.4 Metabolic and biochemical analyses

To monitor non-fasting blood glucose, blood glucose concentration was measured in tail vein blood using an Accu-chek® Active (Roche Diagnostics GmbH, Mannheim, Germany). Glycated hemoglobin (HbA1c) level was assessed in whole blood using a DCA Vantage analyzer (Siemens Medical Solutions Diagnostics, NY, USA).

Blood was collected by cardiac puncture and centrifuged (13,000 rpm for 5 min at 4 °C) to isolate serum, which was aliquoted and frozen at -80 °C until further analysis. Serum concentrations of alanine aminotransferase (Wako Pure Chemical Industries, 9000-86-6) and aspartate aminotransferase (Wako Pure Chemical Industries, 9000-97-9) were determined with commercially available kits. Serum level of total bilirubin (T-BIL) was quantified using a kit (Sekisui Medical, 284-30). DPP-4 activity in plasma was measured using a continuous fluorometric assay with the substrate Gly-Pro-AMC from Bachem (4002520) as previously described [30].

For evaluation of AMC production, plasma sample was added to each well, followed by addition of Gly-Pro-AMC solution as the substrate, with a final concentration of 50 μM in a 50 mM HEPES (pH 7.4) buffer. The enzyme reaction was conducted at room temperature (25 °C). The amount of AMC produced was detected using a FlexStation II384 instrument from Molecular Devices (Sunnyvale, CA, USA). Plasma active GLP-1 was quantified using an ELISA kit (Linco Research, St. Charles, MO, USA) according to the manufacturer's instructions.

### 2.5 Histological analysis

Liver tissues were fixed in 10% buffered formalin for at least 24 h and embedded in paraffin wax for sectioning. Liver sections (4 μm thick) were stained with hematoxylin and eosin (H&E) to assess steatosis and inflammation. Tissue images were captured using an Olympus BX51 microscope (Tokyo, Japan). The NAFLD Activity Score (NAS), a tool to measure changes in NAFLD/NASH during therapeutic trials, was calculated by adding the scores of steatosis, lobular inflammation, and hepatocellular ballooning. Hepatic pathology was determined based on the NAFLD activity score and the NAFLD fibrosis score as previously described [28]. Pathological features were scored as steatosis (0–3), lobular inflammation (0–3), hepatocellular ballooning (0–2), and fibrosis (0–4) by a trained pathologist. In general, NAS equal to or higher than 5 indicates a diagnosis of NASH [31]. Liver fibrosis was assessed by Masson's trichrome staining.

### 2.6 Immunofluorescence staining

Formalin-fixed human liver tissues were embedded in paraffin using standard techniques. The paraffin-embedded human liver tissues were sectioned and blocked with 2% horse serum and then incubated with ULK1 (Cell Signaling, 8054), NLRP3 (Cell Signaling, 15101), or phospho-ACC (S79) (Cell Signaling, 3661) antibody overnight at 4 °C. After incubation, the sections were incubated with secondary antibodies, Alexa Fluor 488 (Invitrogen, A21206) or Alexa Fluor 546 (Invitrogen, A10040), for 60 min at room temperature and mounted with mounting medium (Abcam, ab104139). Fluorescent images were detected with a microscope (BX51TR-32FB3F01, Olympus, Tokyo, Japan).

### 2.6.1 Transmission electron microscopy

Transmission electron microscopy was used to evaluate autophagic vacuoles in the liver tissues of mice. Mouse liver tissues were fixed with a solution containing 2.5% glutaraldehyde and 1% formaldehyde in 100 mM sodium phosphate buffer (pH 7.2) at 4 °C and then postfixed with 2% osmium tetroxide in a 100 mM sodium cacodylate buffer (pH 7.4) for 1 h at 24 °C. The fixed samples were dehydrated in graded alcohol and embedded in epoxy resin (Taab 812 Resin; Marivac Industries, Montreal, Canada). Ultrathin tissue sections (60–70 nm) were stained with uranyl acetate and lead citrate and detected with a transmission electron microscope (Hitachi 7600, Hitachi High-Technologies America, Schaumburg, IL, USA) equipped with a Macrofire monochrome progressive scan CCD camera (Optronics, Goleta, CA, USA) and AMTv image capture software (Advanced Microscopy Techniques, Danvers, MA, USA).

### 2.7 LDH activity assay

HepG2 cells were plated into 96-well plates at a density of 1 × 10^4^ cells/well. The supernatants of HepG2 cells cultured in DMEM or MCD-mimicking media in the absence or presence chloroquine (Sigma Aldrich, C6628) or bafilomycin A1 (Sigma Aldrich, B1793) were collected and transferred into a new 96-well microplate following the manufacturer's instructions (Roche, 11644793001). To measure LDH activity, reaction mixture was added to each well, and the samples were incubated for 30 min at room temperature. The absorbance of the samples was measured at 490–492 nm using a microplate reader (TECAN, Switzerland).

### 2.8 Plasmids and transfection

The myc-hULK1 plasmid was a gift from Do-Hyung Kim (Addgene, 31961). After HepG2 cells were cultured in MCD-mimicking media for 6 h, they were transfected with the myc-hULK1 vector using Lipofectamine 3000 (Invitrogen, LC3000008) for 6 h in the absence or presence of Baf. Expression of ULK1 protein was verified by western blot assay.

### 2.9 RNA interference

siRNAs targeting AMPKα1/2 or ULK1 and negative control siRNA were purchased from Bionia. To reduce off-target effects of siRNA, we pooled two siRNAs targeting the two mRNA types, and then siRNAs and RNAiMAX were diluted in Opti-MED reduced serum medium following the manufacturer's instructions. HepG2 cells were incubated for 16 h with the transfection mixture, with a final siRNA concentration of 100 pM, and then supplemented with fresh medium.

### 2.10 Analysis of autophagy by confocal microscopy

The GFP-LC3 plasmid was purchased from ORIGENE (RC100021). HepG2 cells were transfected with GFP-LC3 vector using Lipofectamine 3000 (Invitrogen, LC3000008) for 24 h. The cells were pretreated with gemigliptin for 6 h and then washed with phosphate-buffered saline (PBS). The cells were incubated in MCD-DMEM with gemigliptin or DMSO for 24 h. Transfected cells were fixed with freshly prepared 4% paraformaldehyde for 10 min and then washed in phosphate-buffered saline. Fixed HepG2 cells were observed using a confocal microscope (Zeiss, Gottingen, Germany).

### 2.11 RNA extraction and qRT-PCR

Total RNA was isolated from mouse liver tissues using an RNeasy Plus Mini Kit (QIAGEN, 74034) and from HepG2 cells using TRIzol (Invitrogen, 15596–018). Total RNA (1.0 μg) was reverse transcribed into cDNA using the High-Capacity RNA-to-cDNA kit (Thermo Fisher Scientific, 4387406) following the manufacturer's protocol. Expression of target genes, *Nlrp3, Caspase-1, Il1b,* and *TNF-a*, was analyzed by quantitative real-time PCR (Roche, LightCycler 480) using SYBR green PCR Master Mix (Life Technology, 4385612). Relative differences in gene expression were determined using the 2^–△△CT^ method. Gene expression was normalized to that of *β-actin*.

### 2.12 Immunoblotting

Protein extraction from FFPE sections was performed using the Qproteome FFPE Tissue Kit following the manufacturer's instructions (QIAGEN, 1042481). Total proteins from mouse liver tissues and HepG2 cells were extracted in PRO-PREP^TM^ protein extraction solution (iNtRON Biotechnology Inc., 17081) with phosphatase inhibitor (Sigma-Aldrich, 4906837001). The concentrations of extracted protein were determined using the BCA Protein Assay kit (Thermo Fisher Scientific, 23225).

Equal amounts of proteins were separated on polyacrylamide gels and electrophoretically transferred onto polyvinylidene fluoride membranes (Millipore, IPVH00010). After blocking, the membranes were incubated with primary antibodies against phospho-mTOR (Cell Signaling, 5536), phospho-AMPK (Cell Signaling, 2535), AMPK (Cell Signaling, 2532), phospho-RPS6KB1 (Cell Signaling, 9208), BECLIN-1 (Cell Signaling, 3495), SQSTM1 (Cell Signaling, 5114), LC3B (Cell Signaling, 3868), ULK1 (Cell Signaling, 8054), cleaved-caspase-3 (cell signaling, 9664), cleaved-caspase-1 (Santa Cruz Biotechnology, sc-56036), IL1β (Santa Cruz Biotechnology, sc-12742), TNFα (Santa Cruz Biotechnology, sc-52748), NLRP3 (Cell Signaling, 15101), phospho-NF-κB (Cell Signaling, 3033), and β-ACTIN (Santa Cruz Biotechnology, sc-47778), followed by incubation with horseradish peroxidase-conjugated anti-mouse IgG (Santa Cruz Biotechnology, sc-516102), anti-rabbit IgG (Santa Cruz Biotechnology, sc-2030), and anti-hamster IgG (Santa Cruz Biotechnology, sc-2789). Bands were detected using an enhanced chemiluminescent detection kit (Amersham, RPN223).

### 2.13 Statistical analysis

Data are reported as mean ± S.E.M. The overall significance of the data was examined by Student's *t* test (with two conditions) or ANOVA, followed by Bonferroni's or Tukey's multiple comparisons test. p < 0.05 indicated statistical significance using Prism 9.0 software (GraphPad Software).

## 3 Results

### 3.1 Inflammasome activity is increased with decreased autophagy-related gene expression in NASH patients

**Figure 1:**
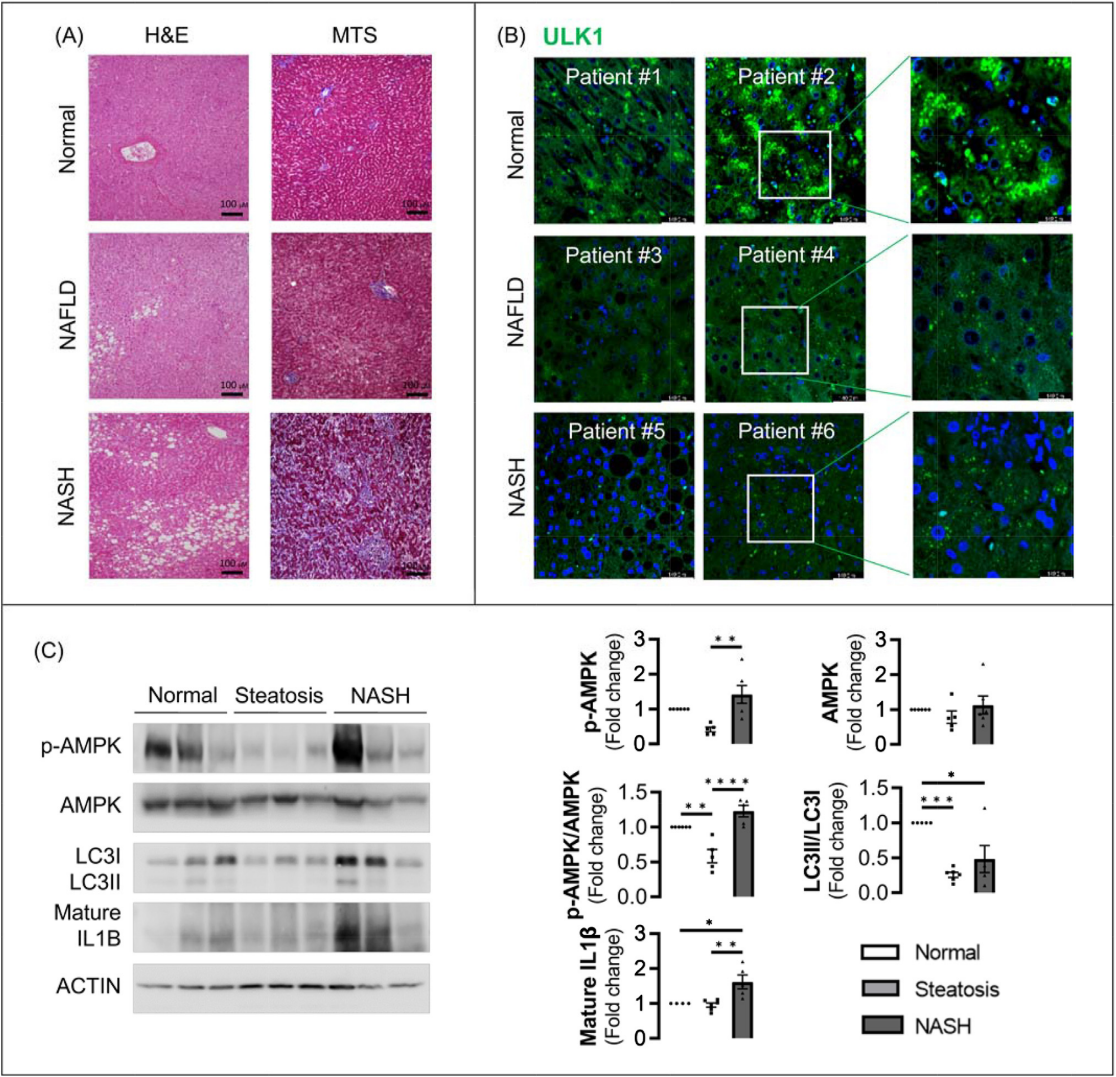
Human NASH showing increased inflammasome activity with reduced autophagy induction and ULK1 expression. (**A**) Representative histological images of human liver sections stained with H&E and Masson's trichrome staining. (B) Immunostaining for pACC (S79) in sections from human patients. (**C**) Representative immunoblot analyses in normal, NAFLD, and NASH liver tissues. Normal (n = 6), steatosis (n = 6), and NASH (n = 6).

Liver histology was performed with hematoxylin and eosin and Masson's trichrome staining to evaluate the status of fatty liver in patients with normal liver, steatosis, and NASH (Figure 1A). Masson's trichrome stained–liver sections revealed fibrosis in the livers of patients with NASH. Reduced expression of ULK1 was confirmed by immunofluorescence and immunoblot assay in liver tissue from patients with steatosis and NASH compared with normal livers (Figure 1B,C). Additionally, we observed a decrease in the LC3II/LC3I ratio in liver tissue from patients with steatosis and NASH compared to normal livers (Figure 1C). As expected, the expression of p-AMPK was decreased in steatosis patients compared with controls. However, in NASH patients, the expression of p-AMPK was restored compared with that of patients with NAFLD. Consistent with these results, the reduced expression of p-ACC (S79) in patients with steatosis was restored in NASH patients to a level comparable to that of the control group (Fig. S1A). These results indicate that downregulation of p-AMPK is not required for inhibition of autophagy in NASH. NLRP3 (Fig. S1B) and mature IL1β levels (Figure 1C) were also elevated in the livers of patients with steatosis and NASH compared with livers from healthy controls.

### 3.2 The parameters of diabetes mellitus were not altered by treatment with gemigliptin in MCD diet–fed mice

We used an MCD-induced NASH model because it does not mimic the human metabolic syndrome characterized by insulin resistance and obesity, and the effect of gemigliptin on NASH rather than its antidiabetic effect can be considered [32,33].

As expected, body weight was not significantly altered by gemigliptin treatment in MCD diet–fed mice, and this trend persisted throughout the treatment period (Fig. S2A). While plasma DPP4 activity in MCD diet–fed mice was significantly increased compared with that in control mice (Fig. S2B), active GLP-1 concentration in MCD diet–fed mice was comparable with the level in control mice (Fig. S2C). However, gemigliptin significantly inhibited MCD diet–induced plasma DPP4 activity (Fig. S2B). Consistent with the marked reduction of plasma DPP4 activity caused by administration of gemigliptin, active GLP-1 content was markedly induced in mice fed the MCD diet with gemigliptin (Fig. S2C). Despite the increase in active GLP-1 in MCD diet–fed mice treated with gemigliptin, the treatment did not affect HbA1c and blood glucose excursion fowling oral glucose challenge (Figs. S2D and S2E). Additionally, glucose-stimulated insulin levels were not different in MCD diet-fed mice with vehicle and MCD diet-fed mice treated with gemigliptin (Fig. S2F). Thus, the marked induction in GLP-1 of gemigliptin-treated mice had no meaningful impact on body weight or glucose metabolism in MCD diet–fed mice.

### 3.3 Gemigliptin alleviates hepatic steatosis and fibrosis in an MCD diet–induced NASH model

**Figure 2:**
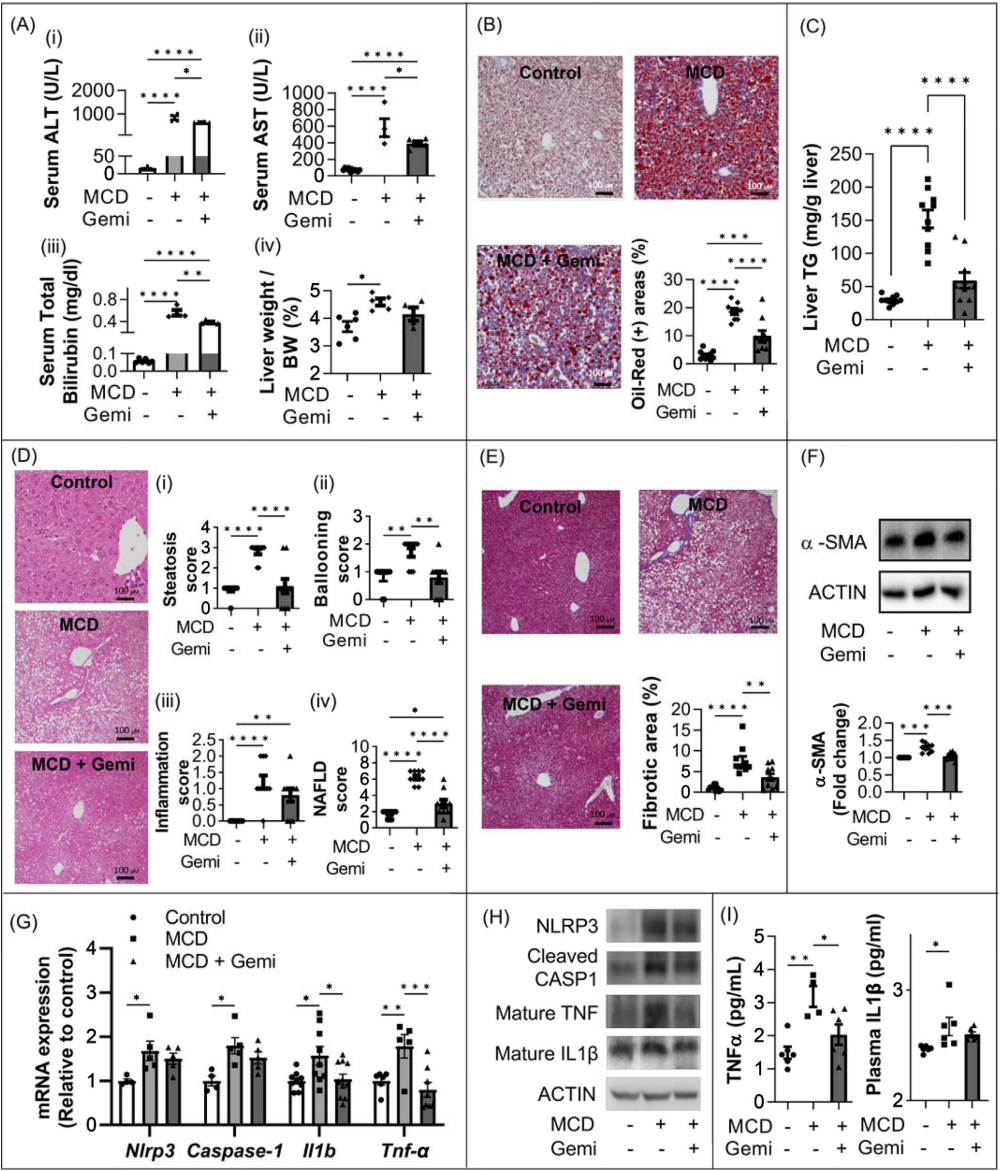
Hepatoprotective effects of gemigliptin on MCD diet–induced inflammation. (**A**) Serum ALT, AST, and total bilirubin levels and liver weight from mice fed either normal chow diet or MCD diet with vehicle or gemigliptin for three weeks. n = 5–10 per group. (**B**) Oil Red O staining and quantification in liver sections. The Oil Red O–positive area was quantified by ImageJ software. (**C**) Hepatic triglycerides (TG). (**D**) Hematoxylin and eosin (H&E) staining of liver sections of experimental mice. The NAFLD activity score (NAS) was measured based on histopathological analysis; steatosis, ballooning, and inflammation. (**E**) Representative Masson's trichrome staining images of liver sections from mice on the MCD diet during three weeks of treatment with vehicle or gemigliptin (0.13% wt/wt). Quantification of Masson's trichrome staining was performed with ImageJ software. (**F**) Liver tissues were subjected to immunoblot analysis. (G) Gene expression of inflammatory markers from mice fed either normal chow diet or an MCD diet with vehicle or gemigliptin for three weeks. (n = 5–8 per group) (H) The expression of inflammasome-related proteins in mouse liver (n = 3–5 per group). (**I**) ELISA of serum TNFα and IL1β in mice treated with MCD diet or MCD + gemigliptin (n = 5–6 per group). Data are shown as mean ± SEM. (∗p < 0.05, ∗∗p < 0.01, ∗∗∗p < 0.001). (For interpretation of the references to colour in this figure legend, the reader is referred to the Web version of this article.)

To examine whether gemigliptin alleviated hepatocellular injury, serum levels of aspartate transaminase (AST), alanine transaminase (ALT), and total bilirubin as well as hepatic triglyceride (TG) contents and histology were assessed in MCD diet–fed mice. Serum levels of ALT and AST were significantly reduced in the gemigliptin-treated group (644.9 ± 28.80 and 390.6 ± 25.04, respectively) compared with the MCD diet–fed group (833.1 ± 87.0; *p < 0.01* and 581.8 ± 109.2; *p < 0.05*) (Figure 2Ai and Aii). The elevated total bilirubin serum level of the MCD diet–fed mice was decreased by treatment with gemigliptin (0.56 ± 0.05 vs. 0.39 ± 0.01; *p < 0.01*) (Figure 2Aiii). Furthermore, liver weight (adjusted by body weight) in the gemigliptin-treated group was significantly lower than that in the MCD diet–fed group (Figure 2Aiv). As shown in Figure 2B, MCD diet–induced hepatic lipid accumulation was decreased by treatment with gemigliptin, as assessed by Oil Red O staining and measured by a quantitative image analyzer. Consistent with this, gemigliptin administration significantly attenuated the MCD diet–induced liver TG increase (Figure 2C). However, there was no significant change in serum TG concentration in MCD diet–fed mice compared with control mice, and serum level of TG was significantly decreased in the gemigliptin-treated group compared to the MCD-diet fed group (data not shown).

Reduction in steatosis, hepatocellular ballooning, and inflammation by administration of gemigliptin were observed in hematoxylin and eosin (H&E) staining (Figure 2D). The NAFLD activity score was significantly decreased in gemigliptin-treated mice compared with MCD diet–fed mice (Figure 2D), as assessed by the NAS system. An increase in fibrotic area was found in the livers of MCD diet–fed mice, whereas treatment with gemigliptin significantly decreased hepatic fibrosis (Figure 2E). Consistent with the significant reduction of hepatic fibrosis by administration of gemigliptin, hepatic expression of α-SMA, a fibrosis marker, was significantly decreased in the gemigliptin-treated group compared with the MCD-diet fed group (Figure 2F). In addition, gemigliptin treatment decreased MCD diet–induced mRNA expression of *Nlrp3, Caspase-1, Il1β,* and *Tnf-α* (Fig. 2G). Hepatic expression of TNFα and IL1Β proteins (Figure 2H) and their plasma levels (Figure 2I) in gemigliptin-treated mice were reduced compared with levels in mice fed the MCD diet. Therefore, gemigliptin treatment improved inflammation in the NASH model.

### 3.4 ULK1 expression and induction of autophagy are decreased in MCD diet–fed mice but are ameliorated by treatment with gemigliptin

**Figure 3:**
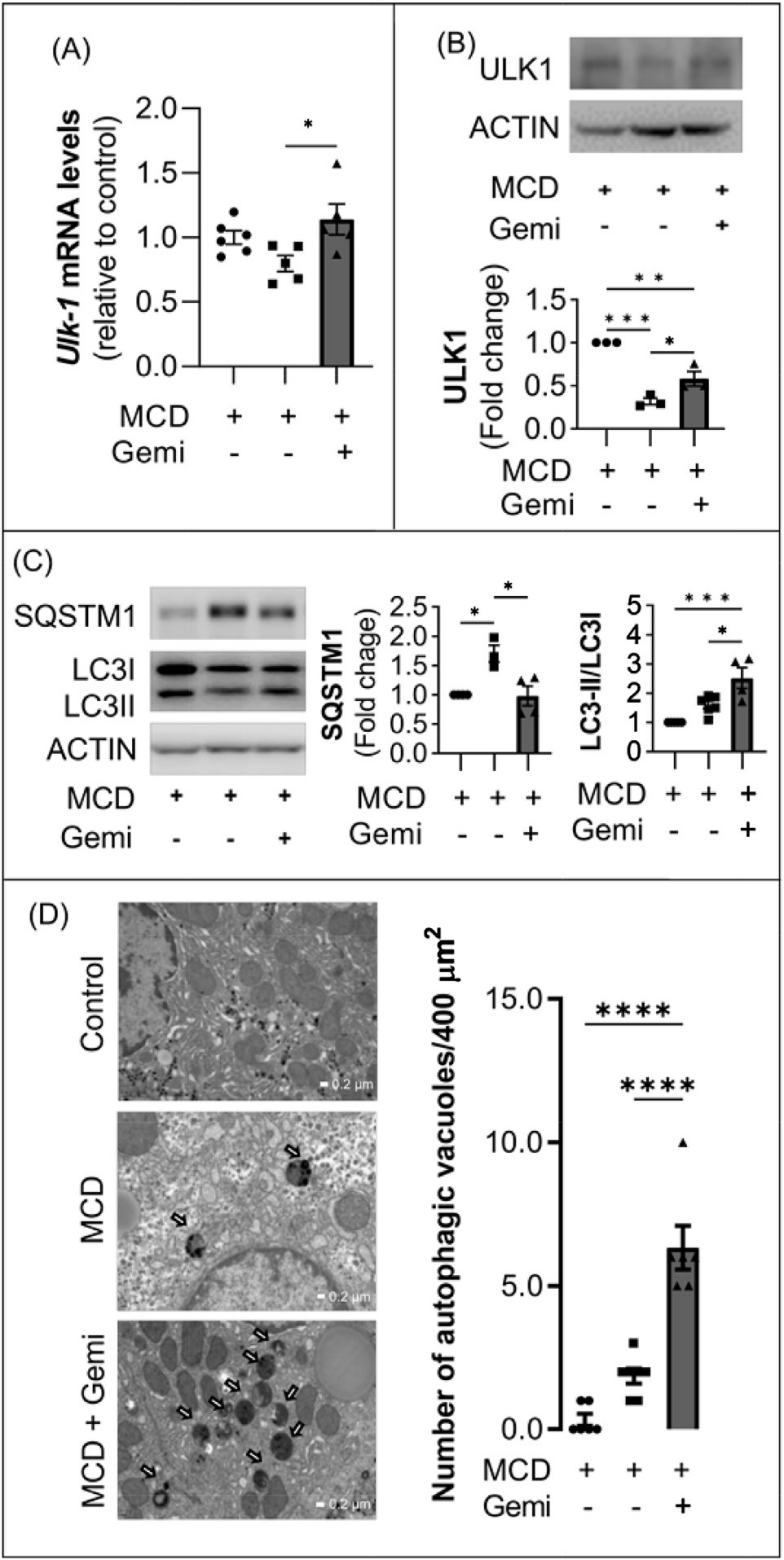
Gemigliptin treatment restored the expression of ULK1 and autophagy in MCD diet–fed mice. (**A**) qPCR and (**B**) immunoblot analyses of mouse liver tissues treated with gemigliptin or vehicle for ULK1. Densitometry analysis of ULK1 expression. (**C**) Representative immunoblot analysis in liver tissues of mice fed an MCD diet with vehicle or gemigliptin. Expression of SQSTM1 and LC3II/LC3I ratio were determined from the optical density–based data of immunoblots. (**D**) Autophagic vacuoles (red arrows) were detected in the electron microscopy images of liver sections of mice. The number of autophagic vacuoles (AVs) per 400 μm^2^ cytoplasm was counted and shown (D, right graph). Data are shown as mean ± SEM of 3–5 mice per group. (∗p < 0.05, ∗∗p < 0.01, ∗∗∗p < 0.001, ∗∗∗∗p < 0.0001).

Previous studies have demonstrated that autophagy plays a key role in treatment of steatosis; these results suggest pharmacological modulation of autophagy as a novel therapeutic avenue for treatment of NASH. To test the hypotheses that hepatic expression of ULK1, one of the most upstream autophagy-related factors, and autophagy induction are suppressed in NASH, and that restoration of ULK1 expression and induction of autophagy can be achieved by treatment with gemigliptin in MCD diet–induced NASH mice, ULK1 and autophagy induction were evaluated in the model. Downregulated hepatic expression of ULK1 transcript as well as ULK1 protein was detected in MCD diet–fed mice compared with control mice, while ULK1 expression was restored in MCD diet–fed mice treated with gemigliptin (Figure 3A,B). In addition, ULK1 expression was decreased in the liver from a Western diet (WD, 40% kcal fat, 20% kcal fructose, 2% cholesterol)-induced NASH model (Fig. S3A). The LC3-II/LC3-I ratio was significantly increased and the expression of SQSTM1 was decreased in MCD diet–fed mice treated with gemigliptin compared with untreated MCD diet–fed mice (Figure 3C). In addition, gemigliptin alleviated MCD diet–induced expression of p-mTOR and p-RPS6KB1 and restored the expression of BECLIN in MCD diet–fed mice (Fig. S4A). However, AMPK expression did not increase after administration of gemigliptin to MCD diet–fed mice. Furthermore, electron microscopy showed that the number of autophagic vacuoles was significantly increased after gemigliptin treatment in MCD diet–fed mice (Figure 3D).

### 3.5 MCD-mimicking media suppresses hepatic expression of ULK1 and autophagy, but gemigliptin ameliorates cell apoptosis by inducing ULK1 expression and autophagy in vitro

**Figure 4:**
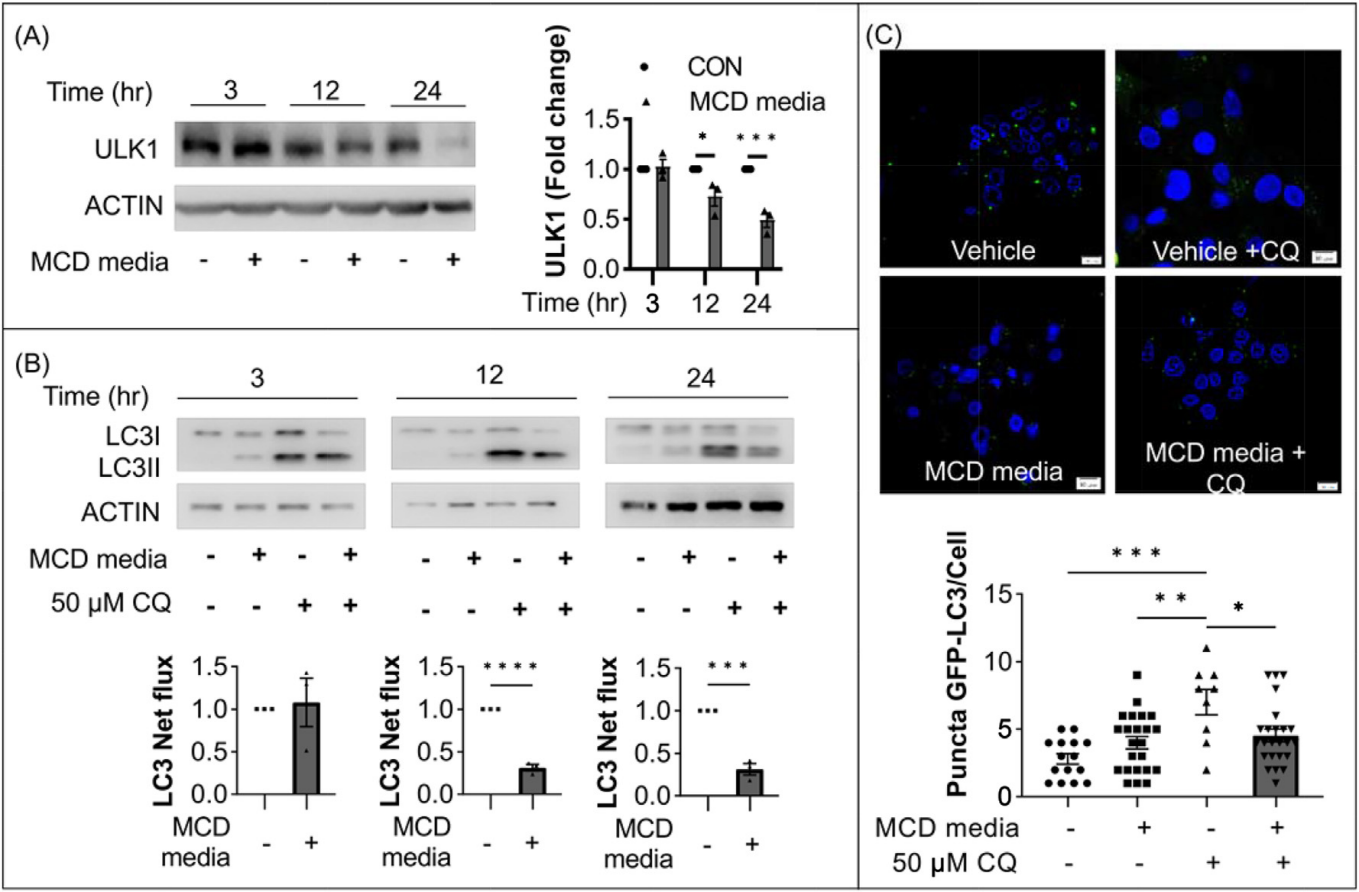
Expression of ULK1 and autophagy machinery was downregulated in HepG2 cells cultured with MCD-mimicking media. (**A**) Representative immunoblot of HepG2 cells cultured in MCD-mimicking media in a time-dependent manner (3, 12, and 24 h). (**B**) Representative immunoblot of HepG2 cells cultured in MCD-mimicking media in the absence or presence of 50 μM chloroquine (CQ) for 4 h. Autophagy flux was determined by subtracting the amount of LC3-II in the absence of CQ from the amount of LC3-II in the presence of CQ for each of the conditions, which was designated as “LC3 net flux” and graphically displayed. (**C**) HepG2 cells were transfected with plasmids driving the expression of GFP-LC3B and then cultured in MCD-mimicking media for 24 h in the absence or presence of 50 μM CQ for 4 h. Fluorescent images were obtained by confocal microscopy. The number of GFP-LC3 puncta was counted and shown. Data are shown as mean ± SEM of three per group. (∗p < 0.05, ∗∗∗p < 0.001, ∗∗∗∗p < 0.0001).

Since ULK1 plays an essential role in initiation of autophagy, we examined whether hepatic expression of ULK1 was suppressed in an in vitro model of NASH. As shown in Figure 4A, HepG2 cells exposed to different culture times (3, 12, 24 h) in MCD-mimicking media showed significantly reduced expression of ULK1 in a time-dependent manner. Furthermore, palmitate decreased ULK1 expression in dose-dependent manner (0, 0.2, 0.5 mM) in HepG2 cells (Fig. S5A).

We then conducted multiple experiments to demonstrate MCD-mimicking media–induced dysfunctional autophagy in vitro. Dysfunctional autophagy was validated with autophagic flux assays; as shown in Figure 4B, accumulation of LC3-II (named LC3 net flux) was reduced in HepG2 cells cultured with MCD-mimicking media compared with the control group. Next, we transfected the GFP-LC3 plasmid into HepG2 cells to evaluate autophagic flux. As shown in Figure 4C, the number of punctate GFP-LC3 structures was decreased in cells in MCD-mimicking media with 50 μM chloroquine compared with the control group.

**Figure 5:**
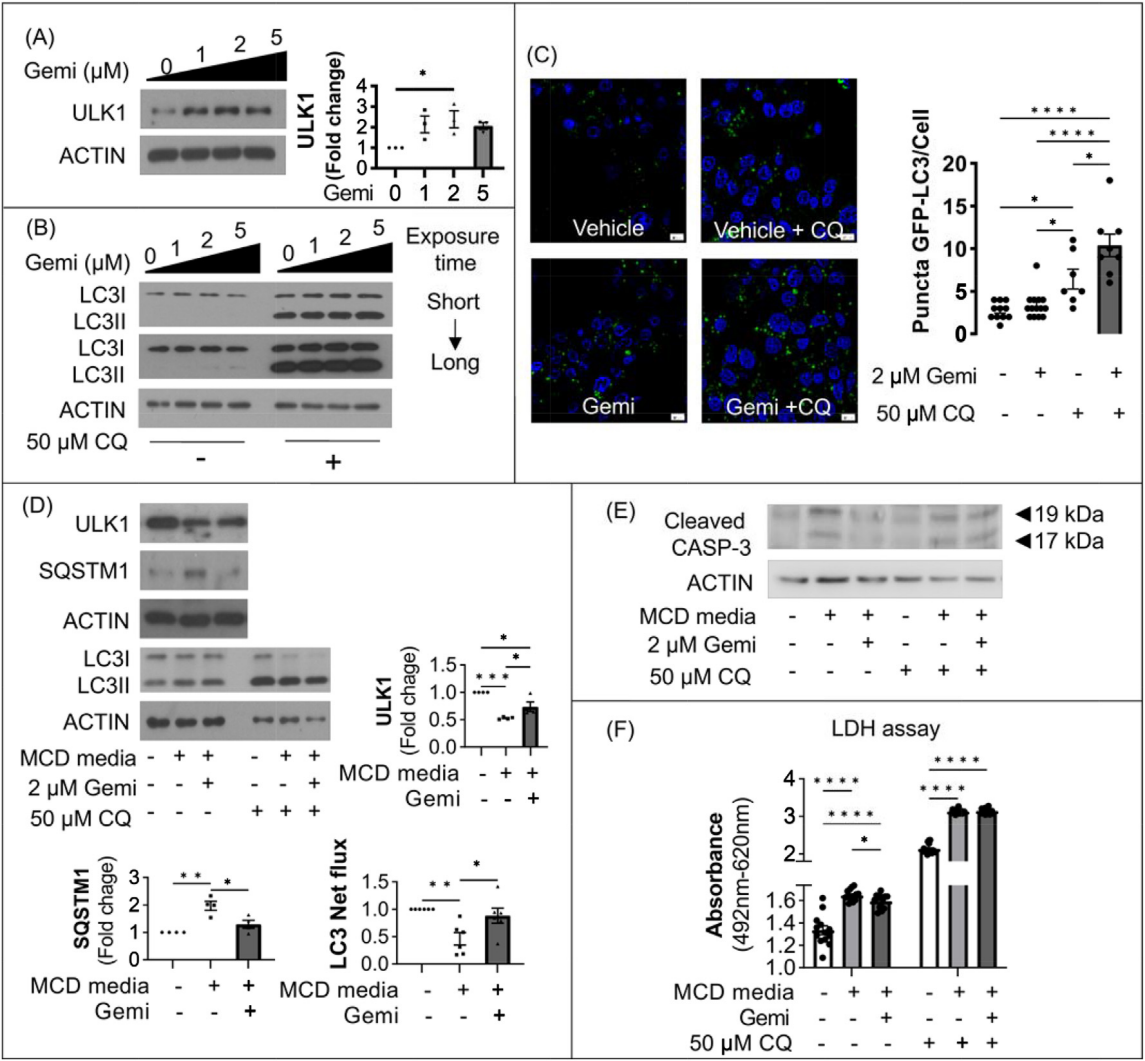
Gemigliptin-induced autophagy ameliorates cell apoptosis in hepatocytes. (**A**) HepG2 cells were treated with various doses of gemigliptin - (0, 1, 2, and 5 μM) for 24 h, followed by immunoblotting. (**B**) HepG2 cells after gemigliptin or vehicle (DMSO) treatment for 24 h in the absence or presence of 50 μM CQ were subjected to immunoblotting. (**C**) HepG2 cells were transfected with the GFP-LC3B plasmid and then treated with 2 μM gemigliptin for 24 h in the absence or presence of 50 μM CQ for the last 4 h. The histogram shows the number of LC3A puncta per cell. (**D**) Representative western blot analyses of HepG2 cells exposed to MCD-mimicking media for 24 h or to 2 μM gemigliptin pre-treatment for 6 h followed by MCD-mimicking media for 24 h in the absence or presence of 50 μM CQ for 4 h. Quantified LC3 net flux is shown. (**E**) CASP3 and (**F**) LDH assay for relative cell survival of HepG2 cells exposed to MCD-mimicking media for 24 h or to pre-treatment with 2 μM gemigliptin for 6 h before MCD-mimicking media exposure in the absence or presence of 50 μM chloroquine (CQ). The data are shown as mean ± SEM. (∗p < 0.05, ∗∗p < 0.01, ∗∗∗p < 0.001, ∗∗∗∗p < 0.0001).

To verify that gemigliptin induced upregulation of ULK1 expression and autophagy induction, HepG2 cells were cultured with different concentrations of gemigliptin. As shown in Figure 5A, different concentrations of gemigliptin (0, 1, 2, 5 μM) significantly induced the expression of ULK1 in a dose-dependent manner. In autophagy flux assays, increased LC3 net flux was observed in gemigliptin-treated HepG2 cells (Figure 5B). This increase in LC3 net flux occurred in a dose-dependent manner. Consistent with these findings, treatment with 2 μM gemigliptin increased the number of GLP puncta in HepG2 cells transfected with plasmid expressing GLP-LC3 in the presence of 50 μM CQ compared with control group (Figure 5C). Furthermore, treatment with gemigliptin prevented MCD-mimicking media–induced suppression of ULK1 expression and LC3 net flux and reduced MCD media–induced SQSTM1 expression in HepG2 cells (Figure 5D). We also found that autophagy regulators (mTOR- RPS6KB1 signaling and p-AMPK) and autophagy markers (BECLIN and SQSTM1) were restored by treatment with gemigliptin (Fig. S6A).

Next, we used chloroquine (CQ), a pharmacological inhibitor of autophagy, to determine whether the beneficial metabolic effects of gemigliptin are mediated by the autophagic process. As shown in Figure 5E, treatment with gemigliptin was no longer able to reduce MCD-mimicking media–induced cleaved caspase-3 in the presence of CQ. Consistent with this result, gemigliptin decreased MCD-mimicking media–induced LDH activity, whereas gemigliptin was unable to attenuate cytotoxicity in the presence of CQ in HepG2 cells (Figure 5F).

### 3.6 Gemigliptin induces autophagy via a ULK1-dependant pathway

**Figure 6:**
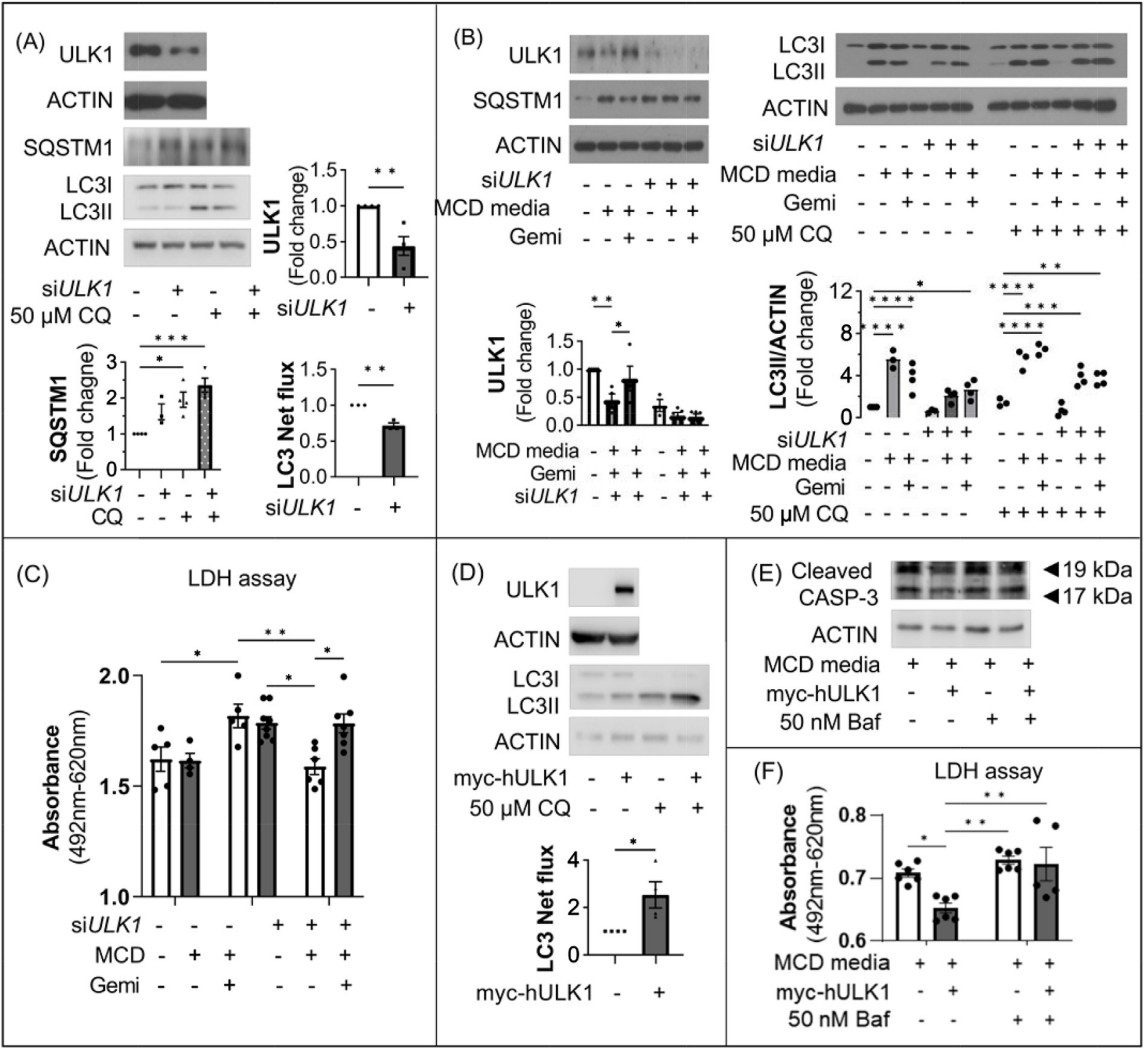
Gemigliptin induced autophagy via the ULK1-dependent pathway. (**A**) Representative immunoblots of HepG2 cells transfected with siRNAs against ULK1 and scramble control for 24 h in the absence or presence of 50 μM CQ for the last 4 h. (**B**) Alleviation of autophagy by gemigliptin was negligible in ULK1-knockdown cells. (**C**) LDH assay was also performed in HepG2 cells transfected with scramble control and siULK1 cultured in MCD-mimicking media for 24 h or 2 μM gemigliptin pre-treatment of 6 h followed by MCD-mimicking media for 24 h. Expression of the indicated proteins was quantified by densitometry. (**D**) Results of immunoblot analysis showed increased LC3 net flux in HepG2 cells transfected with myc-hULK1 vector. (**E**) Representative blot and (**F**) LDH assay showing the rescue effect of Ulk1 overexpression on CASP3 cleavage and cell death in HepG2 cells cultured in MCD-mimicking media in the absence or presence of 50 nM Baf A1 for 6 h, respectively. Data are shown as mean ± SEM. (∗p < 0.05, ∗∗p < 0.01, ∗∗∗p < 0.001, ∗∗∗∗p < 0.0001).

To determine whether gemigliptin induced autophagy through a ULK1-dependent pathway, siRNA directed against ULK1 was transfected into HepG2 cells. As shown in Figure 6A, transfection with *Ulk1* siRNA, which downregulated ULK1 expression, decreased the LC3 net flux and resulted in accumulation of SQSTM1. As shown in Figure 6B, gemigliptin could not restore ULK1 expression after siRNA-mediated knockdown. An autophagic flux analysis using CQ showed that the expression of LC3II in the gemigliptin-treated group was comparable to that in the NASH model group after knockdown of ULK1, indicating that gemigliptin can induce autophagy in the absence of ULK1 expression (Figure 6B).

Next, we examined whether ULK1-mediated autophagy induction was responsible for apoptosis using a vector driving ULK1 overexpression or siRNA directed against ULK1 in HepG2 cells. As shown in Figure 6C, while MCD-mimicking media significantly increased LDH activity in HepG2 cells, gemigliptin significantly decreased cell death. However, upon siRNA-mediated ULK1 knockdown, gemigliptin could not attenuate MCD-mimicking media–induced cell death.

The ULK1 vector increased ULK1 expression and promoted LC3 net flux (Figure 6D). In the absence of Baf, MCD-mimicking media–induced cleaved caspase-3 and LDH were significantly attenuated by overexpression of ULK1. However, in the presence of Baf, MCD-mimicking media–induced cleaved caspase-3 and LDH were not alleviated by ULK1 overexpression (Figure 6E,F). These results indicate that gemigliptin-induced upregulation of autophagy is dependent on ULK1 expression level.

### 3.7 Gemigliptin improves inflammation via the autophagy-mediated inflammasome pathway

We observed that gemigliptin attenuated MCD-induced NASH and induced autophagy. However, the molecular mechanism underlying the anti-inflammatory effect remains uncertain. Hence, we examined the role of gemigliptin in autophagy, which plays an important role in the inflammatory processes that occur in NASH.

**Figure 7:**
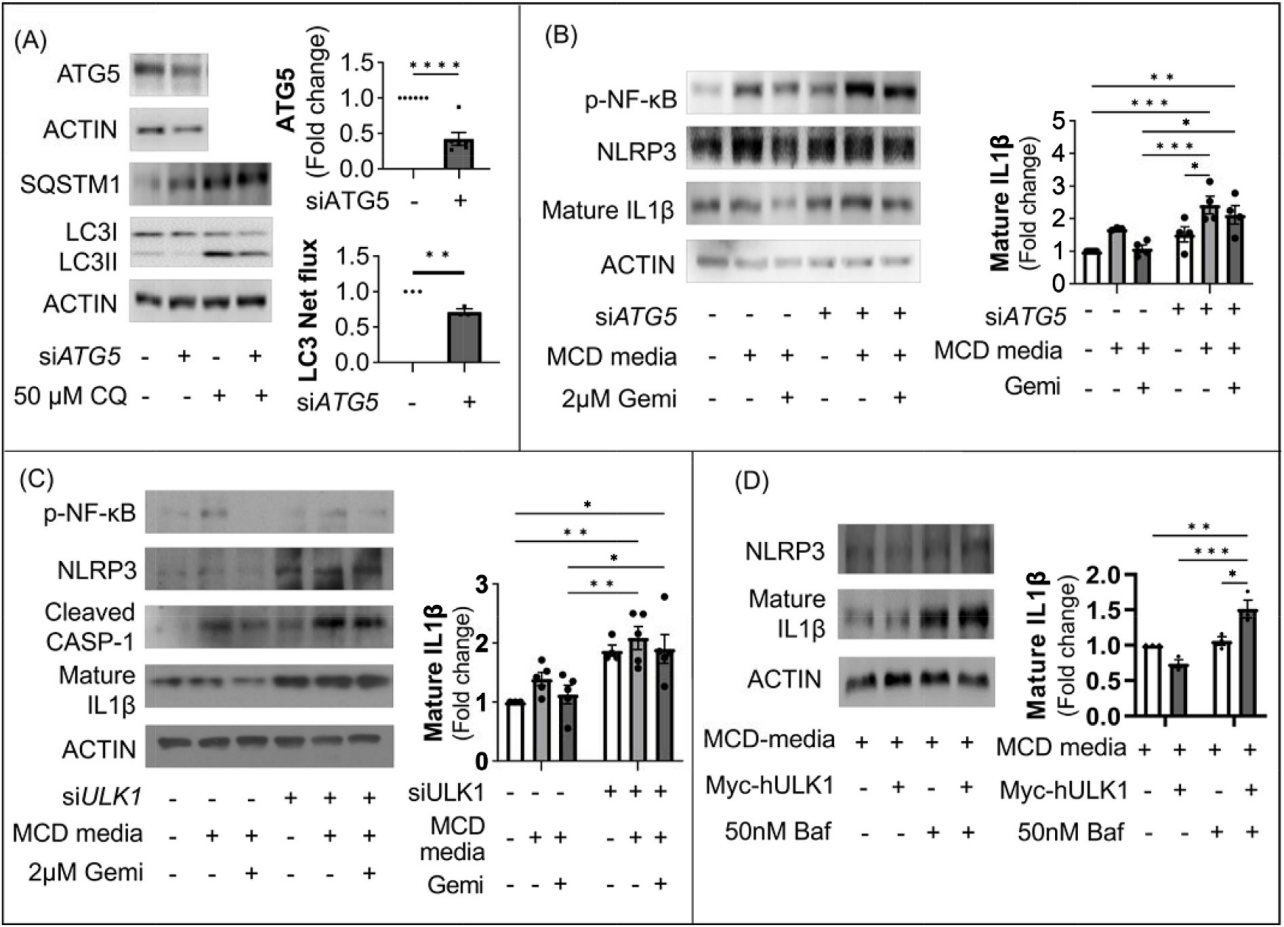
Gemigliptin ameliorates inflammation via the autophagy-mediated inflammasome pathway. (**A**) Representative immunoblots of HepG2 cells transfected with siRNAs against ATG5 and scramble control for 24 h in the absence or presence of 50 μM CQ for the last 4 h. Quantified LC3 net flux is shown. (**B**) The expression of inflammasome-related proteins in scramble control and siATG5 HepG2 cells treated with MCD-mimicking media with 2 μM gemigliptin or vehicle. Quantified mature IL1β is shown. (**C**) Western blots and densitometric analysis of scramble control and siULK1 HepG2 cells. (**D**) Representative blot and densitometric analysis showing the ameliorating effect of Ulk1 overexpression on inflammasome-related protein in HepG2 cells. Data are shown as mean ± SEM. (∗p < 0.05, ∗∗p < 0.01, ∗∗∗p < 0.001, ∗∗∗∗p < 0.0001).

We inhibited autophagy induction by treatment with siRNA directed against ATG5 (Figure 7A). Nuclear factor kappa B (NF-κB)-dependent cytokines IL1β and tumor necrosis factor (TNF) were suppressed by gemigliptin, whereas this effect was blocked by treatment with siRNA directed against ATG5 (Figure 7B). Upon siRNA-mediated knockdown of ULK1, gemigliptin treatment could not reduce expression of MCD-mimicking media–induced p-NF-κB, NLRP3, cleaved caspase-1, and mature IL1 β (Figure 7C). However, MCD-mimicking media–induced mature IL-1 β was alleviated by ULK1 overexpression, whereas inhibition of autophagy using Baf was unable to decrease mature IL-1 β, an inflammasome marker (Figure 7D). These results suggest that gemigliptin ameliorates hepatic inflammation through ULK1-mediated autophagy induction.

### 3.8 Gemigliptin upregulates ULK1-mediated autophagy through an AMPK-independent pathway

**Figure 8:**
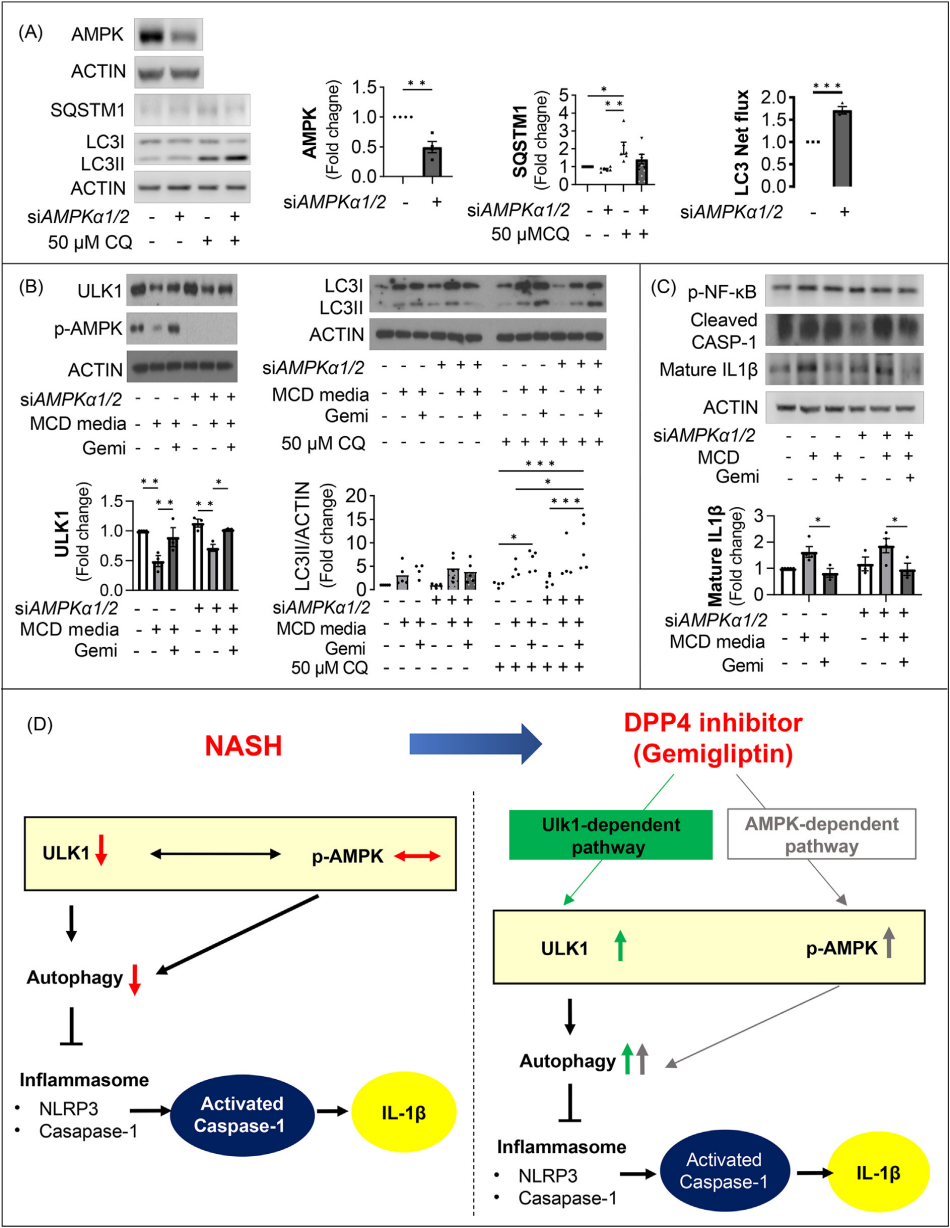
Gemigliptin upregulates ULK1-mediated autophagy through an AMPK-independent pathway. (**A**) Representative immunoblots of HepG2 cells transfected with siRNAs against AMPKα1/2 and scramble control for 24 h in the absence or presence of 50 μM CQ for the last 4 h. Quantified LC3 net flux and SQSTM1 are shown. (**B**) Representative western blots of scramble control and siULK1 HepG2 cells after treatment with MCD-mimicking media for 24 h or 2 μM gemigliptin pre-treatment for 6 h, followed by MCD-mimicking media in the absence or presence of 50 μM CQ for 4 h. The expression of indicated proteins was quantified by densitometry. (**C**) The expression of inflammasome-related proteins in scramble control and siAMPKα1/2 HepG2 cells treated with MCD-mimicking media with 2 μM gemigliptin or vehicle. Quantified mature IL1β is shown. (**D**) A schematic diagram of the proposed mechanism by which gemigliptin ameliorates MCD diet or medium-induced NASH. Data are shown as mean ± SEM of 3–5 per group. (∗p < 0.05, ∗∗p < 0.01, ∗∗∗p < 0.001, ∗∗∗∗p < 0.0001).

As shown in Supplementary Fig. 5, gemigliptin induced AMPK phosphorylation, which plays an essential role in the transcriptional regulation of autophagy. To determine whether gemigliptin induced ULK1 expression via an AMPK-independent pathway, siAMPKα1/2 was transfected into HepG2 cells. As shown in Figure 8A, AMPK was downregulated by transfection with siAMPKα*1/2*, and LC3 net flux was upregulated. Notably, gemigliptin restored MCD-mimicking media–reduced expression of ULK1 as well as LC3 net flux, regardless of the level of AMPK expression (Figure 8B). With respect to the inflammasome, gemigliptin reduced MCD-mimicking media–induced mature p-NF-κb, cleaved caspase-1, and mature IL1β regardless of expression of AMPK (Figure 8C). These findings indicate that the effects of gemigliptin on attenuation of liver inflammation and fibrosis in NASH models is ULK1-mediated autophagy pathway-dependent but is independent of AMPK (Figure 8D).

## 4 Discussion

In this study, we showed the beneficial effects of gemigliptin on amelioration of hepatic inflammation and fibrosis by inducing ULK1-mediated autophagy. This study had three main findings. First, ULK1 expression and induction of autophagy were significantly downregulated in the livers of NAFLD/NASH patients, a NASH murine model, and a hepatic cell line exposed to palmitate or MCD-mimicking media. Second, gemigliptin treatment alleviated hepatic steatosis in association with increasing ULK1 expression and autophagy. Third, gemigliptin treatment restored ULK1 expression and consequently induced autophagy through an AMPK-independent pathway.

Autophagy is an essential mechanism to maintain cellular homeostasis by eliminating damaged or dysfunctional organelles through a lysosomal-dependent pathway [34,35]. The possible involvement of autophagy during the progression and development of nonalcoholic fatty liver disease was first suggested by the discovery that macroautophagy, also known as lipophagy, mediates the breakdown of lipid droplets in hepatocytes, modulating the development of hepatic steatosis [36,37]. Furthermore, autophagy is known to alleviate NASH through inhibition of the inflammasome [20,38,39].

Several preclinical studies have recently shown a potential link between DPP-4 inhibition and autophagy induction. Treatment with sitagliptin ameliorated body weight gain, lipid metabolic disorder, and steatosis as well as hepatic insulin resistance in leptin-deficient *ob/ob* mice by inhibiting inflammatory responses and activating autophagy through the AMPK/mTOR pathway [40].

Another report showed that the reduced autophagic responses in OLEFT after myocardial infarction (MI) were associated with a deficiency in AMPK/ULK1 activation, decreased response of AKT/mTOR/S6 signaling, and increased interaction between BECLIN-1 and BCL-2, which are important molecular events for suppressing autophagy. Interestingly, administration of vildagliptin induced autophagy without normalization of either the AMPK/ULK-1 or mTOR/S6 signaling pathway [41].

On the basis of these reports, we investigated the effect of gemigliptin on autophagy using HepG2 cells cultured in MCD-mimicking medium and mice fed an MCD diet to exclude the possibility of NASH alleviation via metabolic effects such as blood glucose improvement by treatment with gemigliptin. We first demonstrated that gemigliptin ameliorated MCD diet–induced NASH, which is an acquired metabolic disorder characterized by hepatic steatosis, fibrosis, and inflammation. We confirmed that MCD diet–induced hepatic steatosis and ballooning score were attenuated by treatment with gemigliptin based on the NAS system. In addition, we found that treatment with gemigliptin reduced ALT, AST, total bilirubin, and TG contents. Consistent with the marked reduction of hepatic fibrosis by gemigliptin, TNFα and IL1B were significantly lower in the liver and plasma of the gemigliptin-treated group than in the MCD diet–fed group (Figure 2H,I). Although increase in GLP-1 by gemigliptin improved MCD diet–induced NASH, gemigliptin administration in mice fed the MCD diet did not significantly affect body weight, non-fasting blood glucose, or HbA1c. Our findings suggest that gemigliptin treatment improved inflammation in the NASH model without metabolic effects. Similar to our findings, clinical trials evaluating DPP4 inhibitors have reported a significant reduction in fibrosis scores and liver enzymes (AST and ALT levels) in NAFLD patients without diabetes [42,43]. However, there are conflicting reports suggesting that DPP4 inhibitors do not have a beneficial effect on fibrosis and transferases [44,45]. These small-scale clinical trials were conducted in patients with NAFLD for 24 weeks of therapy. Indeed, it has been suggested that observation of such effects may require long-term treatment periods of 1 year or longer [43]. Considering the sample sizes and treatment durations, based on this information, we can postulate that DPP4 inhibitors may improve NAFLD. However, further research with larger sample sizes and longer treatment durations is needed to fully understand the effects of DPP4 inhibitors on NASH.

To investigate the potential molecular mechanisms leading to induction of autophagy by gemigliptin treatment, we evaluated whether the AMPK-ULK1 signaling pathway was involved in this autophagy flux. Notably, we found that autophagy-related proteins, such as ULK1 and LC3II/LC3I ratio, were reduced in NASH patients (Figure 1B,C) and NASH mouse models (Figure 3A–C), whereas the expression of AMPK in NASH groups was similar to that in control groups (Figure 1B and Fig. S4A). These results indicated that autophagy activity was suppressed through the ULK1-mediated pathway regardless of p-AMPK in NASH mouse models and NASH patients. As Boudaba et al. reported that downregulation of AMPK expression does not promote fatty liver development, the AMPK pathway may not be necessary to accumulate lipids in the liver of MCD diet–fed mice [46]. We also observed that expression of ULK1 was decreased in MCD-mimicking media–cultured or palmitate-treated HepG2 cells in a time- or dose-dependent manner, respectively. However, treatment with gemigliptin enhanced ULK1 and AMPK activity, which increased autophagic activity in an in vitro model. Unexpectedly, downregulation of ULK1 expression in MCD-fed mice was restored by gemigliptin treatment, but AMPK expression was not altered. This indicates that the protective effect of gemigliptin on the NASH mouse model, at least in the present *in vivo* study, occurs through an AMPK-independent pathway. Consistent with our findings, previous studies reported that autophagy flux was impaired with decreased hepatic ULK1 activity in HFD-fed mice and human NAFLD patients [20,[47], [48], [49]]. In the same context, suppression of Mir214-3p expression increased autophagy by increasing the expression of ULK1, thereby improving fatty liver disease [47].

To determine whether gemigliptin exerts ULK1-dependent ameliorative effects on autophagy induction, we used siRNA to manipulate the cellular levels of ULK1 and found that gemigliptin was not able to induce autophagy. These results indicate that gemigliptin induces autophagy via a ULK-1-dependent pathway. Unexpectedly, we observed that gemigliptin-induced AMPK was dispensable for autophagy induction. AMPK acts as a sensor of cellular energy that is linked to regulation and induction of autophagy by suppressing mTOR complex 1 (mTORC1) activity or activating ULK1 [50]. However, compound C, an inhibitor of AMPK, induces autophagy through the AKT/mTOR pathway despite inhibition of AMPK activation [51]. Consistent with this observation, metformin, an AMPK inducer, suppresses mTORC1 signaling and induces autophagy in an AMPK-independent manner [52,53]. In addition, AMPK acts to suppress ULK1 activation, resulting in reduction of autophagic activity [54]. Other studies have suggested that ULK1 and autophagy activation occurred independently of AMPK, and that AMPK activation was not observed [[55], [56], [57]]. These results indicate that the increase in phosphorylation of AMPK upon induction of autophagy was not essential and can act as an autophagy suppressor in certain environments. In this study, we suppressed expression of AMPK using siRNA to evaluate whether AMPK activation is required for gemigliptin-induced effects. We found that ULK1 and LC3 net fluxes were increased by gemigliptin treatment even under the conditions of AMPK α1/2 knockdown. From these results, we suggest that gemigliptin induces ULK1 expression through an AMPK-independent pathway, resulting in autophagy induction.

Previous studies have demonstrated that autophagy protects against activation of the disease-causing NLRP3 inflammasome [39,58]. This inflammasome plays an essential role in liver inflammation and fibrosis through autocatalytic cleavage of pro-caspase-1 and maturation and secretion of interleukin-1B [59,60]. In *Nlrp*^−/−^ mice, reduced NLRP3-inflammasome activity inhibits liver damage, whereas inhibition of autophagy stimulates the NLRP3 inflammasome to promote inflammatory states and metabolic disorders [61]. Our data showed decreased expression of NLRP3 and mature IL1β in human NASH patients. In addition, we demonstrated that gemigliptin not only downregulated cleaved caspase-1, but also attenuated mature IL1β via a ULK1 pathway-mediated autophagy-dependent pathway in HepG2 cells and liver of MCD diet–fed mice. These anti-inflammatory effects of gemigliptin are partially consistent with previous reports showing that gemigliptin inhibited the expression of TNFα, IL1β, and IFNγ by blocking NF-κB, JNK, and STAT1/3 phosphorylation [25,62].

Regarding inflammasome attenuation through ULK1-mediated autophagy induction by gemigliptin, ULK1 overexpression reduced mature IL1β in HepG2 cells cultured with MCD-mimicking media. However, ULK1 overexpression was no longer able to reduce mature IL1β in the presence of an inhibitor of autophagy, Baf. Collectively, these data suggest that gemigliptin-induced autophagy via a ULK1-dependent pathway is a potential mechanism by which gemigliptin ameliorates NASH.

One limitation of this study is that the MCD mouse model used in the current study shows suppression of VLDL release, which is one of the hallmarks of defective hepatic lipid metabolism in NAFLD [63]. However, previous studies have demonstrated that that gemigliptin ameliorated NASH in different mouse models, including those induced by high-fat, high-cholesterol- or Western diet-induced [64,65]. Consequently, these previous reports can complement the limitations of the animal model we used. Nonetheless, further studies involving a broader range of animal models are required to extend the therapeutic effects of gemigliptin to humans.

In conclusion, our study found that gemigliptin effectively ameliorates hepatic steatosis, inflammation, and fibrosis in an MCD diet–induced NASH model mice by enhancing autophagy through an increase in ULK1 expression. Therefore, gemigliptin may be a promising therapeutic agent for treatment of NASH.

## Author contributions

Y.S., H. Y., S. K., and C. P designed experiments, interpreted the data, and wrote the manuscript. Y.S., H. Y., J. K, and Y. L. performed the experiments. I. D. provided humans liver samples and pathological examination results. C. P. is the guarantor and the lead contact for this work.

## Data Availability

Data will be made available on request.
