# Supplementary material for: Gemigliptin, a DPP4 inhibitor, ameliorates nonalcoholic steatohepatitis through AMP-activated protein kinase-independent and ULK1-mediated autophagy
Source: Mol Metab. 2023 Sep 20;78:101806. doi: 10.1016/j.molmet.2023.101806 (PMC10542016; doi:10.1016/j.molmet.2023.101806)
Supplement: Multimedia component 1 [file mmc1.doc]

**Gemigliptin, a DPP4 inhibitor, ameliorates nonalcoholic steatohepatitis through AMP-activated protein kinase-independent and ULK1-mediated autophagy**

**Young-Mi Song, Hyekyung Yang, Juhee Kim, Yoonjin Lee, Sung-Ho Kim, In-Gu Do, Cheol-Young Park**

**Table of contents**

Supplementary Figure 1. Human NASH shows increased inflammasome activity with restored pACC (S79) expression.

Supplementary Figure 2. Metabolic effects of gemigliptin on MCD diet–fed mice.

Supplementary Figure 3. Hepatic expression of ULK1 was reduced in Western diet-induced NASH model.

Supplementary Figure 4. Gemigliptin ameliorated the impaired autophagy caused by the MCD diet.

Supplementary Figure 5. Palmitate reduced the expression of ULK1 in a dose-dependent manner as well as LC3 net flux.

Supplementary Figure 6. Gemigliptin ameliorated MCD-mimicking media-reduced autophagy induction in HepG2 cells.

Supplementary Table 1. Information related to the human liver samples analyzed in Figure 1.


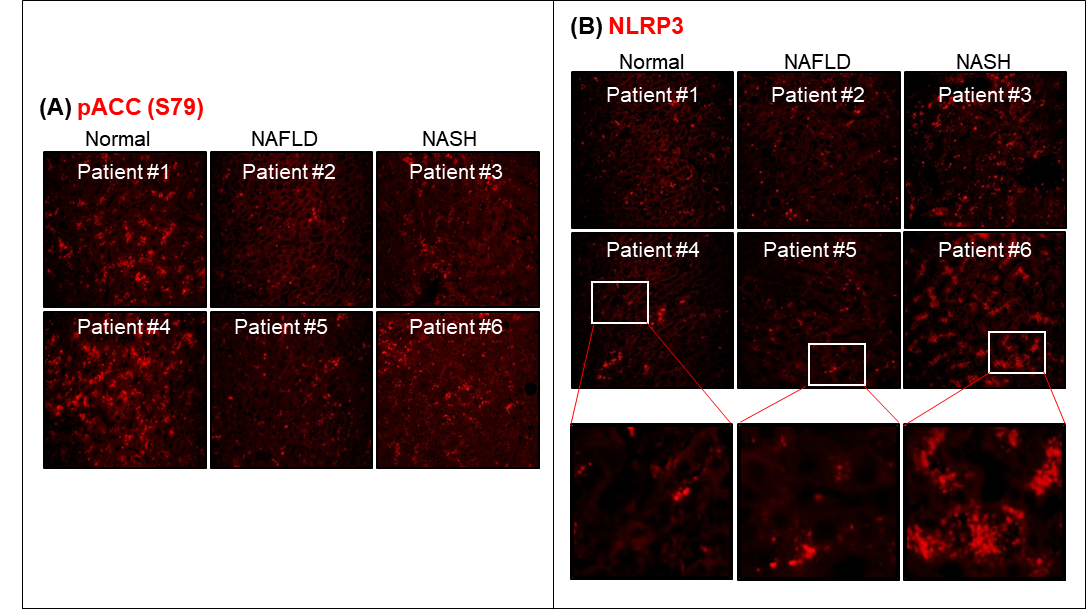
 Supplementary Figure 1

Figure S1. Human NASH shows increased inflammasome activity with restored pACC (S79) expression. Immunostaining for (A) pACC (S79) and (B) NLRP3 in sections from human patients.

Supplementary Figure 2.
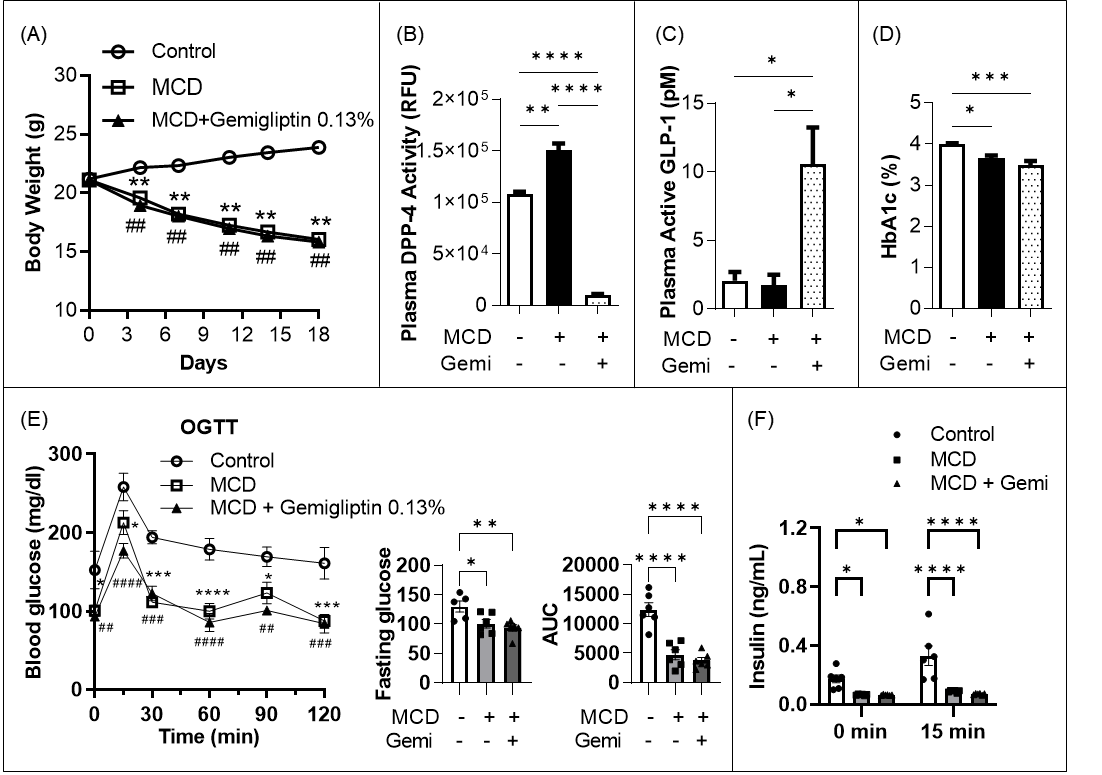


Figure S2. Metabolic effects of gemigliptin on MCD diet–fed mice. C57BL/6N mice were fed an MCD diet containing gemigliptin 0.13% wt/wt for three weeks (n=10 per group). (A) Changes in body weight of mice on the MCD diet during the three weeks of treatment with vehicle or gemigliptin (0.13% wt/wt). **p < 0.01, MCD diet–fed mice vs. control mice; ##p < 0.01, Gemigliptin vs. Control mice. (B) Plasma active DPP-4 and (C) active GLP-1 levels. (D) Levels of HbA1c. (E) Blood glucose and fasting glucose levels after oral glucose (OGTT, 1.5 g/kg) and area under the curve (AUC) for glucose excursion in overnight fasted at 3 weeks after feeding MCD diet or MCD diet containing gemigliptin. (F) Plasma insulin measured before and 15 min after oral glucose challenge. *p < 0.05, **p < 0.01, ****p < 0.0001, MCD diet–fed mice vs. control mice; ###p < 0.001, ###p < 0.0001, Gemigliptin vs. Control mice. (F) Plasma insulin before and 15 min after an oral glucose challenge. Data are shown as mean ± SEM (*p < 0.05, **p < 0.01, ***p < 0.001, ****p < 0.0001).

Supplementary Figure 3


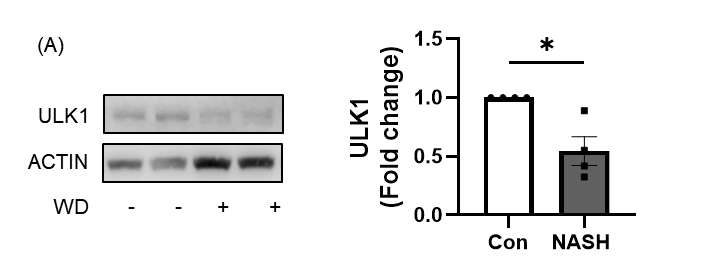


Figure S3. Hepatic expression of ULK1 was reduced in a Western diet-induced NASH model. (A) Immunoblots for ULK1 and ACTIN in liver from mice fed normal chow diet or Western diet (WD, 40% kcal FAT, 20% kcal Fructose, 2% Cholesterol) for 17 weeks. Quantified ULK1 expression was graphically displayed. . Data are shown as mean ± SEM of 4 mice per group. (*p < 0.05)

Supplementary Figure 4


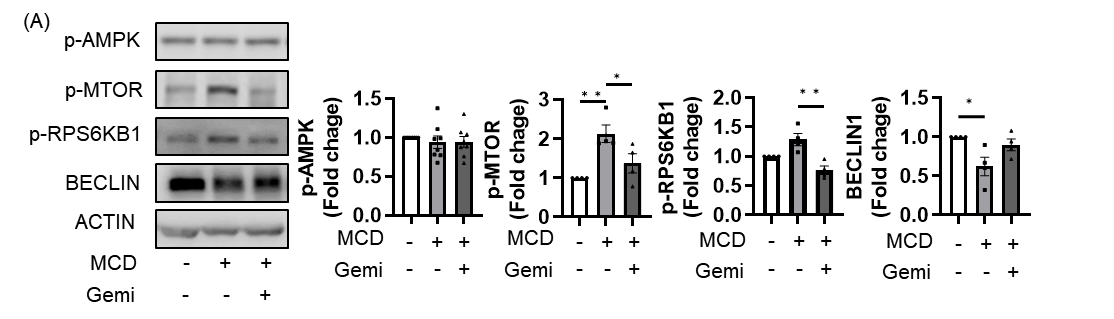


Figure S4. Gemigliptin ameliorated the impaired autophagy caused by the MCD diet. (A) Representative immunoblot analysis in liver tissues of mice fed MCD diet with vehicle or gemigliptin. Expression of each target protein were graphically displayed from the optical density-based data of immunoblots. Data are shown as mean ± SEM. (*p < 0.05, **p < 0.01)

Supplementary Figure 5


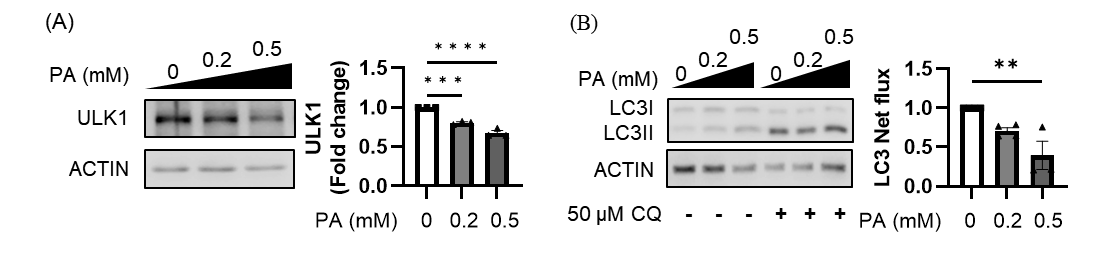


Figure S5. Palmitate reduced the expression of ULK1 in dose-dependent manner as well as LC3 net flux. (A) Expression of ULK1 and (B) LC3I/II in HepG2 cells treated with palmitate (0, 0.2, 0.5 mM) or vehicle for 24 hr in the absence or presence of 50 uM CQ for 4 hr. Quantified ULK1 and LC3 net flux were graphically displayed. Data are shown as mean ± SEM of 3-4 per group. (**p < 0.01, ***p < 0.001, ****p < 0.0001)

Supplementary Figure 6


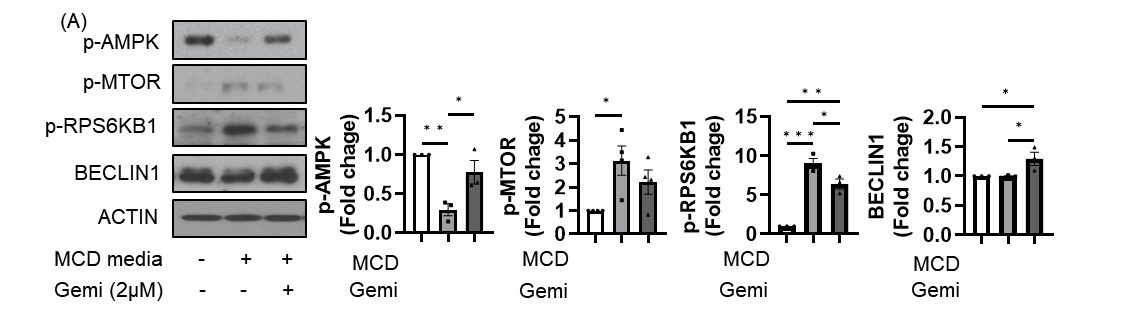


Figure S6. Gemigliptin ameliorated MCD-mimicking media-reduced autophagy induction in HepG2 cells. (A) Representative immunoblot analysis in HepG2 cells cultured with MCD-mimicking media for 24 hr or 2 μM gemigliptin pre-treatment of 6 h followed by MCD-mimicking media for 24 h. Quantified target proteins were graphically displayed. Data are shown as mean ± SEM of 3-4 per group. (*p < 0.05, **p < 0.01, ***p < 0.001)

Supplementary Table 1


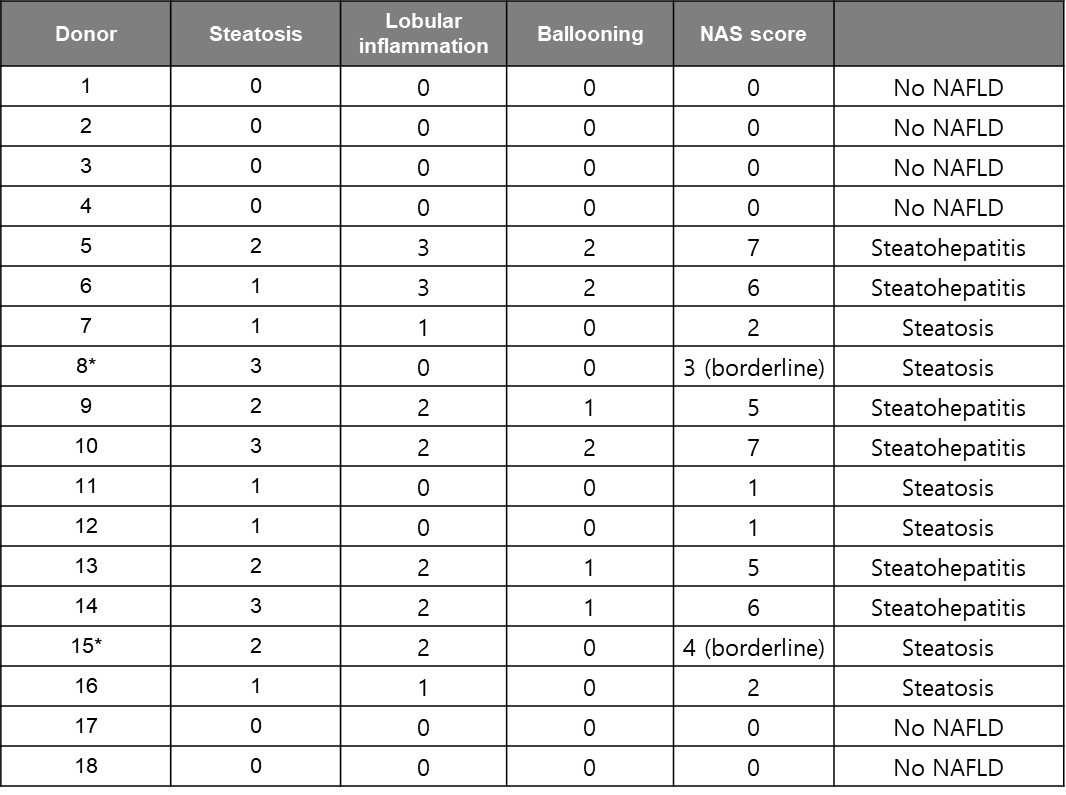


Table S1 Related to Figure 1

Information related to the human liver samples analyzed in Figure 1.

Asterisk (*) indicates that minimal portal fibrosis was observed in the liver tissues of the donors.
